# Correlates of patients’ satisfaction with antenatal care services in a tertiary hospital in Abakaliki, Ebonyi State, Nigeria

**DOI:** 10.11604/pamj.2020.37.342.17925

**Published:** 2020-12-15

**Authors:** Chidebe Christian Anikwe, Chinedu Chukwuemeka Ifemelumma, Kenneth Chinedu Ekwedigwe, Cyril Chijioke Ikeoha, Ogah Emeka Onwe, Ugochukwu Uzodimma Nnadozie

**Affiliations:** 1 Department of Obstetrics and Gynecology, Federal Teaching Hospital, Abakaliki, Ebonyi State, Nigeria,; 2 Department of Paediatrics, Federal Teaching Hospital, Abakaliki, Ebonyi State, Nigeria,; 3 Department of Surgery, Federal Teaching Hospital, Abakaliki, Ebonyi State, Nigeria

**Keywords:** Antenatal care, quality of care, satisfaction, Abakaliki

## Abstract

**Introduction:**

antenatal care is a specialized pattern of care organized for pregnant women to improve their chances of a safe delivery. Assessment of patients' perception of healthcare services is one of the ways of measuring the quality of healthcare as satisfied patients are likely to come back for the services they need and to recommend the services to others.

**Methods:**

this is a cross-sectional study. Two hundred and eighty-four booked antenatal attendees were randomly selected at the antenatal clinic of Federal Teaching Hospital, Abakaliki in November 2016 and interviewed using semi-structured questionnaire. Items in the questionnaire included sociodemographic and obstetric variables, assessment of amenities, total time spent, services and level of satisfaction. Data obtained were analyzed using Epi info TM 7.1.3.10 and presented with a simple percentage and chi-square. Main outcome measure: satisfaction with antenatal care.

**Results:**

the mean age of the respondents was 28.2 ± 4.2 years, majority 130 (45.8%) were within the 25-29 age bracket. Most had tertiary education (146, 51.4%) and less than 10% are grand multipara. In general, 89.4% of the respondents were satisfied with the quality of antenatal care services. Majority of the respondents 170 (59.9%) were unsatisfied with the water supply while 128 (45.1%) were unsatisfied with cervical cancer prevention discussion during the health talk. The mean total time spent in the antenatal clinic was 4.1 hours ± 1.2 hours (range 2-7 hours). Being married and multiparous significantly affected satisfaction with the quality of antenatal care services as represented by P-value of 0.015 and 0.005 respectively.

**Conclusion:**

majority of pregnant women were satisfied with the care they received. Health providers should, however, improve the state of sanitary facilities and ensure the provision of adequate information on cervical cancer screening during health talks.

## Introduction

One of the goals of an obstetrician is to deliver a healthy baby to a healthy and satisfied mother. Antenatal care (ANC) is one of the important instruments in achieving this goal. ANC describes specialized medical care given to pregnant women during pregnancy [1]. It is a preventive health care program aimed at enabling pregnant women to attain and maintain a state of good health throughout pregnancy so as to improve their chances of having safe delivery [2]. It provides opportunities for health education and counseling of the client and her partner on pregnancy, labor and postnatal care, screening and management for pre-existing and any on-going disease in the woman during pregnancy, screening for congenital abnormalities and commencement of preventive health care program for the women [3]. It is one of the pillars of safe motherhood that has contributed to the reduction of maternal and fetal morbidity and mortality [4,5].

Globally, the maternal mortality ratio has been reported to be 216 per 100,000 deliveries with 303,000 maternal deaths occurring in 2015 [6]. The majority (99%) of these deaths occur in developing countries with sub-Saharan Africa accounting for 66% (201,000 maternal deaths) of the global estimate of developing countries [7]. The high maternal [8] and perinatal [9] mortality rates that were reported in Nigeria is unacceptably high and ensuring access to good-quality maternal and child health care could prevent most of these deaths [4,5,9]. A joint statement by the Royal College of Obstetrics and Gynaecology (RCOG) and British Maternal-Fetal Medicine Society (BMFMS) has highlighted the relationship between poor feto-maternal outcomes and women not accessing antenatal care in a timely manner [10].

The determinants of antenatal care are well documented by previous studies in Nigeria and late antenatal booking is a common finding in these studies [11-13] even for those women that had pregnancy complications in their previous confinement [11]. It has been documented also that the majority of pregnant women prefer to deliver outside the hospital in sub-Saharan Africa. Some cogent reasons have been adduced in these studies to account for this unwelcomed finding but we are forced to ask: what is the influence of quality of care received in previous confinement to this finding? Unbooked pregnant women or late booking connotes obstetrics danger [13,14]. Quality of health care is seen as a factor closely related to effectiveness, compliance and continuity of care [15]. Quality of healthcare services can be assessed either objectively or subjectively or by assessing the supply and demand components of health services. Subjectively, assessment of patients´ perception of healthcare services is one of the ways of measuring the quality of healthcare [16].

Patient satisfaction of health services is difficult to define/measure and compare as it is influenced by client and hospital characteristics. It could be defined as an individual's positive evaluation of distinct dimension of health care [17] or as an individual's state of been contented with the care provided in the health system. Lari *et al*. also defined patient satisfaction as the extent of an individual's experience compared with his or her expectations or what patients and the population as a whole desire to receive from health care services [18].

Satisfaction is a multidimensional construct built on the tripod of structure, process and outcome according to Donabedian framework [19]. The framework emphasizes client satisfaction as an outcome of client perception of the physical environment of the hospital, availability of drugs and other consumables, knowledge and attitude of health workers, time spent in receiving care, cost of care and above all the delivery of a healthy baby to a healthy mother. It is not important whether the patient is right or wrong but what is important is how the patient felt. Demographic characteristics such as age, ethnicity, marital status, duration of pregnancy, the frequency of antenatal visits and socio-economic status have been shown to have limited impact on pregnant women´s perception of the quality of antenatal care [20]. Patient with higher satisfaction are more likely to stick to medical recommendations and are likely to come back for the services they need and to recommend the services to others. Measurement of client satisfaction helps in auditing and improvement of care. It involves the description of health care service from the patient's perspective; providing an opportunity for its measurement and evaluation as a function of patients´ satisfaction. Various factors including the attitude of staff, cost of care, the time spent at the hospital and doctor communication have been found to influence patients´ satisfaction in previous studies [15]. It is important for reproductive health care providers to get feedback from women regarding satisfaction with reproductive health services.

To the the best of our knowledge there is a dearth of information about client satisfaction of antenatal care services in the tertiary hospital in Nigeria and efforts are being made by various bodies in Nigeria to make health institutions client-oriented. This study is embarked upon to help fill a knowledge gap since this type of evaluation has not been carried in our center and it is aimed at determining the level of satisfaction and its determinants among antenatal attendee in Federal Teaching Hospital Abakaliki (FETHA). Findings would provide feedback to health care providers, local administrators, hospital managers and policymakers in order to promote patients' satisfaction.

## Methods

**Study area:** Ebonyi State is one of the five states in the south-east geopolitical zone of Nigeria. It has a population of about 3 million people and is inhabited mainly by the Igbos. Most of the populace are rural dwellers and farmers. Federal Teaching Hospital Abakaliki (a tertiary institution) is located at the center of the state and receives referrals from all parts of the state and neighboring states of Abia, Benue, Cross River and Enugu.

The antenatal clinic holds daily from Mondays through Fridays, so also the booking and postnatal clinics. The clinics are run by consultant obstetricians with their teams of resident doctors in the department of the obstetrics and gynaecology. This cross-sectional analytic study was conducted at the antenatal clinic of the Federal Teaching Hospital, Abakaliki in November 2016. In 2015, clinic attendance was 18731 (daily average of 130 women). There are 5 antenatal clinics per week (Monday to Friday), along with booking and postnatal clinic. The clinic usually commences with an interactive health talk co-ordinated by a qualified nurse which usually lasts for at least 60 minutes. The health talk usually covers various topical issues including nutrition, diet, personal hygiene and danger signs in pregnancy, the labor experience, care of the newborn, exclusive breastfeeding and immunization. Other health issues such as hypertension, diabetes mellitus, malaria, anemia, HIV/AIDS and family planning are also discussed. Routine services following the health talk include weight and height measurement, blood pressure estimation, urinalysis, hemoglobin estimation. Thereafter, patients are called individually to see their doctors for clinical examination and treatment. During the consultation, the woman is counseled on birth preparedness and complication readiness by their doctors and their complaints managed. Folic acid, ferrous sulfate, intermittent prophylactic treatment (IPT) and multivitamin supplementation are given. Anti-retroviral therapy and septrin are also given to HIV positive pregnant women. Ethical approval for the study was obtained from the Research and Ethics Committee of the hospital (FETHA/REC/VOL 1/ 2014/190).

**Study instrument:** the study instrument is a pretested questionnaire, adapted from previous studies [15,21] with modification which is divided into sections: socio-demographic and obstetric characteristics; assessment of services provided at antenatal facility, assessment of amenities and health topics discussed at facility, assessment of attitude of the health workers and the hospital physical environment, waiting time/total time spent and overall rating of antenatal care services. Each questionnaire took 10-15 minutes to complete. The satisfaction related variables came up with a high internal consistency (Cronbach`s alpha= 0.82). Total time spent was defined as time spent from arrival at the ANC clinic to the end of the clinic consultation by the obstetrician; while waiting time is defined as time spent from the end of the health talk to the beginning of the clinic consultation. The study population was selected using the ballot method of simple sample method at the ANC clinic after written consent was obtained. They were interviewed by resident doctors in the department of obstetrics and gynaecology who were trained to administer the questionnaire. Translation to native languages was done in cases where the respondents were uneducated.

The client assessment of antenatal care experience was evaluated using four-point Likert scale of strongly agree, agree, disagree and strongly disagree. Those that strongly agree or agree were grouped as satisfied with care while those that disagree or strongly disagree were classified as dissatisfied. To assess women´s overall satisfaction with the quality of antenatal care, the summary section of the questionnaire contained three indicators which included one direct and two indirect summary questions asked against the background of women´s responses to previous inquiries on the various aspects of antenatal care quality. It would be expected that this “overall satisfaction” variable would reflect women´s overall perception of the quality of antenatal care received. This variable was determined by respondents´ affirmative answers to these three questions: “would you register at the facility again? Would you recommend the facility to somebody else?” and “in general, are you satisfied with antenatal care you have received so far in this clinic?”. For the purpose of this study, an affirmative answer to all of the three questions by the respondent was considered an index of true satisfaction with the quality of antenatal care received (satisfaction index).

**Sample size determination and data analysis:** the sample size was calculated using the formula for cross-sectional study:

$$N=\frac{Z^{2}XPXQ}{D^{2}}$$

where, N=required sample size; Z=1.96 at confidence level at 95%; P = 0.81, estimated patient satisfaction from similar studies [15]; D: margin of error at 5% (standard deviation of 0.05) and Q=1-P. A total sample size of 284 was gotten after addition of 20% attrition rate. The data was collected on the five clinic days, Monday through Friday. The data obtained were analyzed using the Epi Info™ 7.1.3.10. The results were presented as percentages and frequencies. Categorical variables were compared with the chi-squared test (χ2). The level of significance was set at P<0.05.

## Results

A total of 284 antenatal clinic attendees were interviewed and used for analysis. In general, 254 (89.4%) of the respondents were satisfied with the quality of the antenatal care services; 268 (94.4%) would likely register at the facility again, while 266 (93.7%) would recommend the facility to someone else. As shown in Table 1, the majority of the respondents, 130 (45.8%) were between 25-29 years old, 268 (94.4%) were married, 182 (64.1%) were para 1-4. Igbo women made up 91.6% (260) of the respondents while 51.4% (146) had tertiary education.

**Table 1:** socio-demographic characteristics of the respondent

Characteristics	Frequency (n)	Percentage (%)
**Age**		
20-24	54	19.0
25-29	130	45.8
30-34	60	21.1
≥35	40,284	14.1
**Marital status**		
Single	16	5.6
Married	268	94.4
**Nature of family**		
Monogamous	270	95.1
Polygamous	14	4.9
**Parity**		
Nullipara	74	26.0
1-4	182	64.1
≥5	28	9.9
**Level of education**		
Primary	42	14.8
Secondary	96	33.8
Tertiary	146	51.4
**Husband's occupation**		
Skilled	122	43
Semi-skilled	86	30.3
Unskilled	76	26.7
**Tribe**		
Igbo	260	91.6
Yoruba	16	5.6
Hausa	8	2.8
Total	284	100

Majority of respondents 170 (59.9%) were unsatisfied with the water supply in the antenatal care. Environmental cleanliness, hygiene (toilet facility) and examination room privacy were considered satisfactory in 244 (85.9%), 168 (59.2%) and 218 (76.8%) of women respectively. Amongst the health topics discussed, prevention of cervical cancer had the highest number of unsatisfied respondents of 128 (45.1%), while the majority, 260 (91.5%) were satisfied with childcare and breastfeeding. On diet and nutrition, danger signs of pregnancy, breast self-examination and HIV information and counseling; 244 (85.9%), 258 (90.8%), 210 (73.9%) and 248 (87.3%) were satisfied respectively (Table 2). On the quality of the consultation process, 254 (89.4%) and 258 (90.8%) were satisfied with history taking and counseling for risk factors. Less than eighty percent (226 (79.6%)) of respondent assessed the registration processes as satisfactory, while 234 (82.4%) were satisfied with the environmental conditions. Record retrieval was rated as unsatisfactory by 88 (31%) of the respondents (Table 2).

**Table 2:** assessment of amenities, health topics, consultation process and services at the ANC facility

Variables	Satisfied n (%)	Not satisfied n (%)
**Amenities**		
Water supply	114 (40.1)	170 (59.9)
Hygiene (toilet facility)	168 (59.2)	116 (40.8)
Electricity supply	280 (98.6)	4 (1.4)
Ventilation	240 (84.5)	44 (15.5)
Sitting arrangement and spacing	224 (78.9)	60 (21.1)
Consultation room privacy	220 (77.5)	64 (22.5)
Examination room privacy	218 (76.8)	66 (23.2)
General environment cleanliness	244 (85.9)	40 (14.1)
**Quality of consultation process**		
Patient history taking	254 (89.4)	30 (10.6)
Patient's examination	252 (88.7)	32 (11.3)
Counseling for risk factors	258 (90.8)	26 (9.2)
Explanation about treatment	236 (83.1)	48 (16.9)
**Health topics**		
Diet and nutrition	224(85.9)	40 (14.1)
Danger signs of pregnancy	258 (90.8)	26 (9.2)
Birth preparedness	256 (90.1)	28 (9.9)
Child care and breastfeeding	260 (91.5)	24 (8.5)
Family planning and child spacing	248 (87.3)	36 (12.7)
Prevention of STI	238 (83.8)	46 (16.2)
HIV information and counselling	248 (87.3)	36 (12.7)
Prevention of cancer of the cervix	156 (54.9)	128 (45.1)
Breast self-examination	210 (73.9)	74 (26.1)
**Quality of consultation process**		
Patient history taking	254 (89.4)	30 (10.6)
Patient's examination	252 (88.7)	32 (11.3)
Counseling for risk factors	258 (90.8)	26 (9.2)
Explanation about treatment	236 (83.1)	48 (16.9)
**Services provided**		
Registration	226 (79.6)	58 (20.4)
Record retrieval system	196 (69.0)	88 (31.0)
Laboratory services	200 (70.4)	84 (29.6)
Pharmaceutical services	224 (78.9)	60 (21.1)
Environmental conditions	228 (80.3)	56 (19.7)
Cost of ANC services	234 (82.4)	50 (17.6)

Two hundred and twenty-four (78.9%) rated the attitude of nurses as good while 276 (97.2%) rated the attitude of doctors as good. The mean time spent during each clinic visit was 4.1 hours ±1.2 (range 2-7 hours). One hundred and ninety - four (68%) of the respondents spent over 3 hours which only 13 (6.7%) of them were not satisfied with ANC services and would likely not register in the facility again. Following cross-tabulation with the Pearson Chi-square test, only marital status, parity and the attitude of nurses and doctors were significantly associated with the rating of ANC services (Table 3).

**Table 3:** cross-tabulation of the overall rating of antenatal services and socio-demographic variables

Variable	Satisfied	Not satisfied	P-value
**Age**			
20-24	47	7	0.06
25-29	120	10	
30-34	53	7	
≥35	32	8	
**Marital status**			
Married	248	20	0.015^*^
Single	12	4	
**Nature of family**			
Monogamous	244	12	0.22
Polygamous	20	4	
**Parity**			
Nullipara	65	9	0.005^*^
1-4	170	12	
≥5	21	7	
**Level of education**			
Primary	42	0	0.26
Secondary	85	11	
Tertiary	134	12	
**Tribe**			
Igbo	242	18	0.18
Yoruba	16	0	
Hausa	8	0	
**Doctors' attitude**			
Good	204	36	0.001^*^
Poor	0	8	
**Nurses' attitude**			
Good	208	16	0.001^*^
Poor	35	25	
**Total time spent**			
≤3 hours	83	7	0.74
>3 hours	181	13	

* Significant

## Discussion

Patient satisfaction is considered an essential component of healthcare services evaluation and an indicator of the quality of healthcare. This study examined the quality of antenatal care services rendered in tertiary health care centers in Abakaliki as perceived by women using this facility for their antenatal care. Generally, most, 254 (89.4%) of the respondents were satisfied with the quality of the antenatal care services. The high level of satisfaction noted in this study agrees with the findings of other similar studies in Nigeria [15,16,22]. Our study showed that more than ninety percent of the respondent would likely register at the facility or recommends the facility to another woman for antenatal care. This agrees with the earlier finding in Lagos, Nigeria [23]. Even though women may generally express satisfaction with the quality of antenatal services despite inconsistencies between received care and their expectations of the facility, the high rate of satisfaction and respondent wish to seek for care in our facility is encouraging. This might help reduce the adverse maternal and child health indices in the area of study [24] and Nigeria in general. Antenatal care could lower maternal deaths [25] and it also provides a window of opportunity to women for receiving other reproductive health services.

In this study, marital status and parity were found to be significantly associated with a higher level of satisfaction and the willingness to register again in the facility. This finding as it directly relates to satisfaction is in support with a study that was based on a recent Nigerian demographic health survey which reported that marital status and increasing parity are positively associated with ANC satisfaction in Nigeria [26]. It has been shown that partner support and previous maternal health care experiences affect a patient´s decision to utilize a health care facility [27]. Oladapo *et al*.found that increasing parity was associated with an increased likelihood of positive perception of antenatal care quality while age, being married, ethnicity, educational level and frequency of antenatal care visits were not associated with overall satisfaction with antenatal care quality [20].

Doctors and nurses are considered ideal for impacting technical knowledge, providing emotional support and assisting in decision making, reassurance and alleviation of anxiety [28]. Good provider-patient relationships are therapeutic and have been described as the single most important component of good medical practice. The majority, 97.2% and 79% of respondents in this study rated the attitude of the doctors and nurses as good respectively; hence, it is not surprising that this had a significant association with the overall level of satisfaction recorded (P=0.001). This high rate could be expected in the study area as it is a teaching center and health professional are expected to demonstrate standard practice. This is consistent with the findings from other similar studies [16,23,28]. The attitude of health workers influences the level of patients´ satisfaction as Ekott *et al*.recorded that the unfriendly attitude of health workers resulted in patients´ dissatisfaction with the services offered [22]. A recent study in Africa by Do *et al*. has shown that antenatal attendees in hospital setting wait longer before receiving anticipated care and this has a detrimental effect on client satisfaction [29]. Long waiting time is also observed in our study but unlike in Do *et al*. [29] study, respondent satisfaction with ANC services is not significantly associated with the women waiting time (OR: 0.852 95%CI 0.328 - 2.213). Apart from the difference in the study setting and design which might account for this finding, it seems that the respondents in our study are able to endure longer if the attitude of health care personnel is deemed positive. This highlights the importance of encouraging positive patient-health worker relationship in promoting patient satisfaction and use of care [30].

A disturbing finding in this study was that 128 (45.1%) of the respondents were dissatisfied with the discussion on cervical cancer prevention during the health talk sessions. This dissatisfaction with cervical education among the respondent is not in consonance with the overall good knowledge of nurses about cervical cancer in the study area who are expected to inform the antenatal attendee [31]. The finding is similar to the findings by Nwaeze *et al*. [15]. This is highly unacceptable as an opportunity to create awareness of the burden of a potentially preventable cancer of the cervix is lost. Cervical cancer still remains a significant cause of mortality among women in developing countries [32] and this need to be addressed by the relevant authorities and more effort should be made in disseminating information on the importance of such screening. Fortunately, the hospital has a very functional “well woman centre” which offers such screening and effort should be made to create awareness among antenatal attendee. Compared to other amenities, women were least pleased with the water supply, toilet facilities and consultation room privacy. It is quite unacceptable as these are a structural element that affects overall patient satisfaction with care [30].

## Conclusion

The index study has shown a high level of satisfaction with the quality of antenatal care services among pregnant women attending the antenatal clinic at FETHA. Only marital status, parity and the attitude of nurses and doctors were significantly associated with the rating of ANC services. The level of satisfaction with cervical cancer information is low and effort should be made to improve this as cervical cancer contributes significantly to cancer-related death in our environment. Most respondents were willing to register again at the facility. However, women´s expression of satisfaction may not indicate that all the elements of care are completely good or in accordance with standards. There is scope for further improvement and we recommend focused group discussion as this may be necessary to gain insight on ways to improve the services. Also, toilet facilities that meet minimum standards should be provided, problems with the water supply should be addressed and the provision of information on cervical cancer screening should be included as an integral part of health talks.

**Limitations of the study:** the study was conducted among women who are already seeking care in the facility and may not reflect the opinion of those who stopped seeking care in the health facility as a result of their dissatisfaction and also the possibility of interviewers´ bias cannot be totally ruled out as women may feel that the study was an audit process conducted by higher authority and thus responded in favor of the health facility in fear of indicting the personnel at the center.

### What is known about this topic

- Antenatal care is an important tool in safe motherhood;
- Multi-dimensional factor influencing maternal satisfaction with antenatal care and how these factors can influence subsequent obstetrics behavior;
- The dearth of knowledge of determinants of antenatal care satisfaction in a tertiary hospital in Nigeria.

### What this study adds

- This study has shown that client satisfaction with antenatal care services in the facility is good and it is positively associated with the client´s marital status, parity and the attitude of nurses and doctors;
- This study thus emphasizes the importance of positive client-provider interaction in improving antenatal care usage. A satisfied mother is likely to use and recommend a facility for antenatal services which is essential in proactively reducing the bad obstetric outcome;
- Our study also highlighted the poor client´s satisfaction with information on cervical cancer and its prevention. This is worrisome as ANC offers a veritable opportunity to educate women on this disease which contributes significantly to the death of women in Nigeria. It calls for auditing of care with training and retraining of antenatal managers on cervical cancer so as to improve information delivery.
